# Higher intensity exercise after encoding is more conducive to episodic memory retention than lower intensity exercise: A field study in endurance runners

**DOI:** 10.1371/journal.pone.0308373

**Published:** 2024-09-13

**Authors:** Roger Makepeace, Michael Craig

**Affiliations:** Department of Psychology, Faculty of Health and Life Sciences, Northumbria University, Newcastle upon Tyne, United Kingdom; University of Tsukuba, JAPAN

## Abstract

An acute bout of exercise in the moments after learning benefits the retention of new memories. This finding can be explained, at least partly, through a consolidation account: exercise provides a physiological state that is conducive to the early stabilisation of labile new memories, which supports their retention and subsequent retrieval. The modification of consolidation through non-invasive exercise interventions offers great applied potential. However, it remains poorly understood whether effects of exercise translate from the laboratory to naturalistic settings and whether the intensity of exercise determines the effect in memory. To this end, adult endurance runners were recruited as participants and completed two study sessions spaced two weeks apart. In each session, participants were presented with a list of words and asked to recall them on three occasions: (i) immediately following their presentation, (ii) after a 30-minute retention interval, and (iii) after 24 hours. Crucially, the 30-minute retention interval comprised our experimental manipulation: higher intensity exercise (running) in the first session and lower intensity exercise (walking) in the second, both completed in a naturalistic setting around participants’ existing physical activity training programmes. Exertion was recorded through heart rate and rate of perceived exertion data. Alertness, mood, and arousal ratings were also collected before and after the 30-minute retention interval. Immediate memory for the two wordlists was matched, but participants retained significantly more words after 30 minutes and 24 hours when encoding was followed by higher than lower intensity exercise. Exertion data revealed that participants experienced vigorous and light exercise in the higher and lower intensity conditions, respectively. Significant improvements in alertness, mood, and arousal were observed following both exercise conditions, but especially in the higher intensity condition. These outcomes reveal that experiencing higher intensity physical activity in the field is conducive to declarative memory retention, possibly because it encourages consolidation.

## Introduction

New memories are fragile upon their formation and vulnerable to disruption [1]. For labile memory traces to be transferred to a more enduring and stable form that can be used adaptively to guide behaviour, they must undergo a process of consolidation [1–3]. This memory stabilisation is proposed to occur through two interrelated processes: (i) early cellular-level consolidation, which is a hippocampal-dependent process that strengthens synaptic connectivity through the induction of long-term potentiation (LTP) in the immediate aftermath of encoding [4, 5] and (ii) later systems-level consolidation, which involves the strengthening of connections between the hippocampus and neocortical regions, facilitating the transition of memory representations into a hippocampal-independent state over the longer term [3, 6].

Contemporary theories propose that (at least) cellular consolidation is an automatic and opportunistic process that occurs especially during states of reduced sensory processing and physical activity, which would otherwise interfere [7–9]. This opportunistic-consolidation hypothesis is supported by (i) behavioural evidence demonstrating superior memory following periods of sleep and quiet rest [10–16], and (ii) neuroimaging and neuroscientific evidence demonstrating a greater magnitude of consolidation-related brain activity (e.g., neural “replay” of recently encoded memories) during periods of quiescence [12, 17–20]. Indeed, the disruption of such mechanisms, for example, through pharmaceutical interventions and periods of task engagement, is found to be detrimental to memory retention [16, 21, 22], especially in those with impaired memory systems [23–25]. These findings evidence the malleable nature of early consolidation processes, where the fate of new memories can be determined in the minutes immediately following their creation.

Converse to observations of superior memory following post-encoding quiescence [e.g., wakeful rest; 16], acute exercise in the post-encoding period can also be conducive to declarative memory consolidation [26–35]. A meta-analysis of published work determined that acute exercise during the consolidation period after encoding positively influences the retention of new episodic information, relative to control conditions including no exercise [36]. Importantly, the effect of post-encoding exercise is dissociable from the influence of exercise in encoding, for example, when exercise is experienced prior to or during the encoding of information [32]. Indeed, current data suggest that there is no additive benefit of experiencing both pre- and post-encoding acute exercise [37]. Still, it is possible that post-encoding exercise could positively affect both the consolidation *and* retrieval of encoded traces should a bout of acute exercise occur shortly following encoding and soon before memory is probed. However, a beneficial effect of acute exercise on consolidation can be isolated, for example, in cases where exercise occurs shortly following encoding, but recall takes place after an extended period (e.g., 24 hours) by which point residual effects of exercise (e.g., on underlying neurophysiology or state of arousal) would be expected to have elapsed [36].

To achieve a positive effect in memory, the duration of the acute post-encoding bout of acute exercise need not be excessive in duration: both short (e.g., 5 minutes) and longer-duration (e.g., 60 minutes) bouts of acute exercise can positively influence consolidation [38, 39], and these effects are not transient but long lasting and detectable at least 24-hours post-encoding [34, 35, 40]. Moreover, in keeping with the time-dependent nature of consolidation, such effects are not restricted to exercise in the immediate aftermath of encoding; an acute bout of exercise can benefit consolidation even when it occurs several hours after encoding [28, 31]. This resonates with the outcomes of the aforementioned meta-analysis, which observed an overall positive effect of acute exercise during early *and* late consolidation [36]. Intriguingly, the meta-analysis also revealed that the effects of exercise during consolidation appear to be moderated by a range of factors, including exercise intensity. Specifically, pooled evidence suggests that vigorous-intensity exercise is beneficial to consolidation, while light and moderate intensity are not [36]. This does, however, conflict with some data suggesting that higher-intensity exercise has minimal effect on consolidation when it occurs soon after encoding [41], possibly because of the induction of an excessive stress response in cases of high-intensity exercise, which is known to be detrimental to consolidation [42]. It is worth noting that research exploring the contribution of exercise intensity to consolidation remains in its infancy and few studies have compared the effects of lower and higher intensity exercise on consolidation directly, especially within naturalistic settings.

Rather than a reduction in interference [e.g., from sensory processing associated with task engagement; 16, 43], post-encoding acute exercise may benefit consolidation because it stimulates neurophysiological activity in the hippocampus and associated networks [26], including the induction of LTP and synaptic plasticity [44]. While the acute cerebrovascular response to a single bout of exercise–and the relationship with cognition–is poorly characterised [45], emerging evidence suggests that even a short period of moderate aerobic exercise lasting ~15–20 minutes can increase hippocampal blood flow significantly [46, 47]. There is also compelling evidence for a positive relationship between acute (and chronic) exercise and the stimulation of brain-derived neurotrophic growth factor (BNDF) [48, 49], which is a precursor of LTP and synaptic plasticity [50], and is implicated in the success of consolidation [51–53]. Independent studies have shown that acute bouts of moderate-intensity [54, 55] and high-intensity [40, 55–57] exercise encourage BNDF expression and support memory retention, but findings are mixed and further work is required to better characterise the specific contributions of exercise duration and intensity to BNDF expression and memory consolidation. Nevertheless, it is possible that neurophysiological and cerebrovascular dynamics might explain, at least partly, why higher intensity exercise in the post-encoding period is reported to benefit early consolidation, relative to light intensity exercise and control (e.g., wakeful rest) conditions [36].

Are there applied consequences for the benefit of acute exercise on consolidation? There is great promise in recommending exercise as a non-invasive intervention to encourage consolidation, which, in addition to the memory benefit, could bring broader benefits in cognition and mood [58, 59]. Hitherto, most studies examining the effect of acute exercise on consolidation have done so within laboratory-based settings using student populations [30]. Despite obvious advantages of laboratory studies, including robust experimental control, such investigations lack the rich contextual features of naturalistic settings. Therefore, for exercise to be recommended as a memory-based intervention, there is a need for empirical studies in naturalistic settings [26]. If successful, there could be scope for exercise to be a recommended consolidation intervention, which may be especially beneficial for those with compromised memory [e.g., older adults; 40] and clinical populations [e.g., those with depression; 60] who may be unable to engage sufficiently with existing invasive (e.g., therapeutic) and non-invasive (e.g., quiet rest) interventions designed to improve memory retention. Thus, there is a need for further evidence demonstrating that an acute bout of exercise can enhance consolidation within naturalistic settings. To achieve this, further insights regarding the intensity of exercise required to achieve a positive effect in memory are required. Given that current evidence [36] indicates that vigorous-intensity exercise benefits consolidation, while light-intensity does not, it is logical to compare the effects of these two exercise intensities on consolidation in a natural setting as a first step towards establishing exercise as a non-invasive memory-promoting intervention.

To this end, the current study aimed to establish whether higher-intensity (vigorous) exercise encourages declarative memory consolidation in naturalistic settings, relative to lower-intensity (light) exercise. This was investigated through a laboratory-based consolidation paradigm [10, 15, 16] that was modified for delivery in the field, where, to promote ecological validity, it was moulded around the normal exercise activities of adult endurance runners. In keeping with contemporary theories and evidence [e.g., 10, 28], it was predicted that participants should demonstrate superior memory retention when encoding is followed by higher intensity exercise than lower-intensity exercise.

## Materials and methods

### Ethics statement

This research was approved by the Faculty of Health and Life Sciences’ Research Ethics Committee at Northumbria University (Ref: 49278). Informed written consent was acquired from participants following an initial study briefing and procedures adhered to the appropriate ethical principles for research in humans.

### Participants

An a priori sample size calculation conducted using G*Power 3.1 [61] indicated that a minimum sample of 34 participants was required to detect a significant within-subject difference in a paired samples t-test (two tailed) when considering 80% power, an alpha level of .05, and a medium effect size (d = 0.5). The chosen effect size resonated with existing laboratory-based work that used similar paradigms, where large effect sizes are often observed [e.g., 10]. A more conservative medium effect was used in the current study given that it was conducted in the field, where extraneous variables likely influenced data to a degree. The minimum sample required (n = 34) was exceeded through the recruitment of 35 adults aged 18 years and older (females: n = 16, males: n = 19; mean age = 48.14 years, SD = 8.78, age range: 29–70 years). All individuals had normal or corrected-to-normal visual acuity, no known premorbid psychiatric or neurological disorders, and were amateur endurance runners recruited from an athletics club in the North East of England, UK. Endurance runners were recruited for the current study to ensure the study could be delivered in a naturalistic setting and that the physical demands of our post-encoding delay conditions (lower vs. higher intensity exercise) could be delivered safely. For example, these individuals were already familiar with the Rate of Perceived Exertion scale [RPE; 62], which was used to guide participants in achieving the desired physical activity exertion level in the two delay conditions–see Procedure. Study recruitment occurred from October to December 2022 and collected data were accessed for analyses following completion of data collection, i.e., from December 2022.

### Design

**Fig 1:**
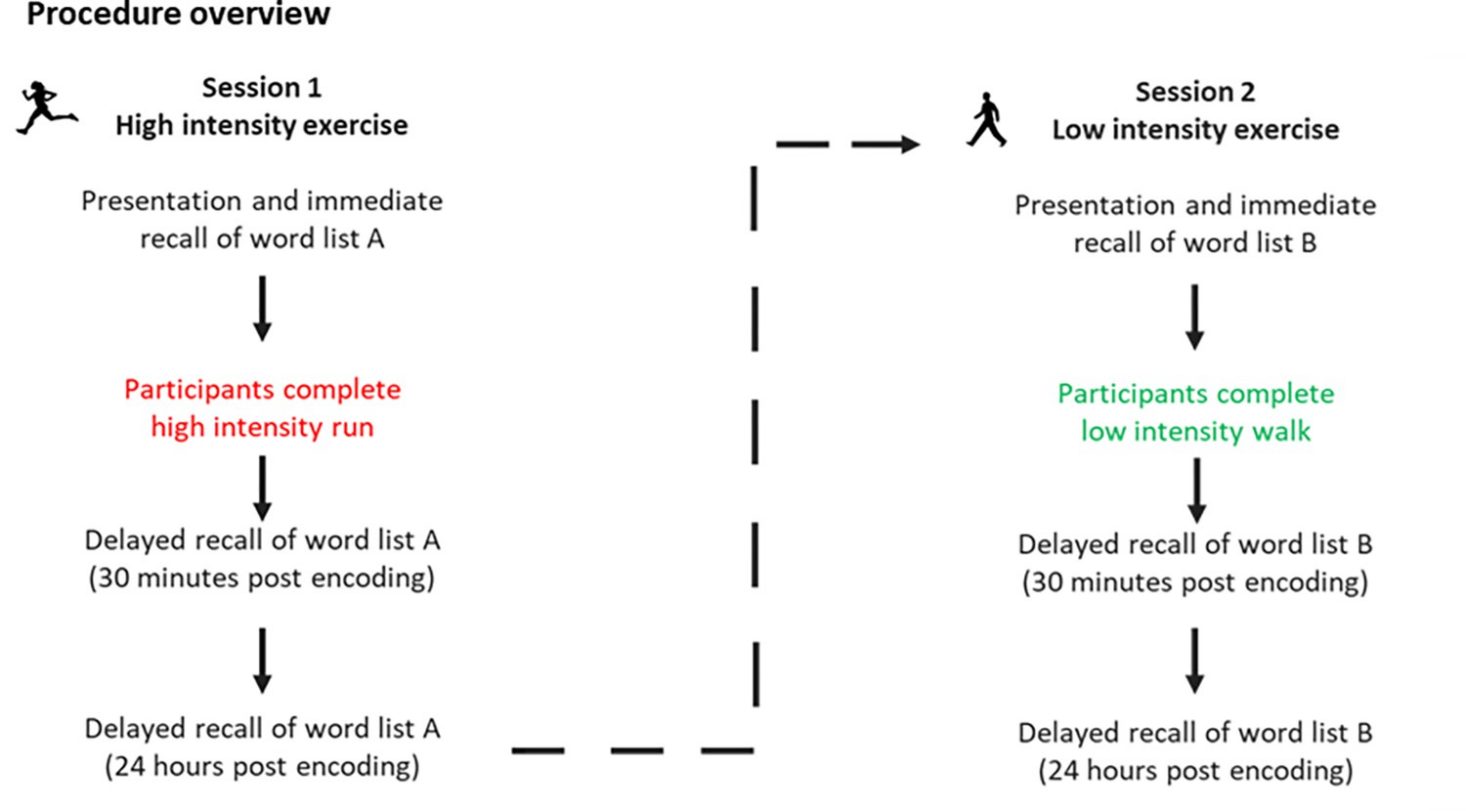
Study procedure. Each participant completed two sessions spaced two weeks apart. In each session, participants completed an episodic memory test where they were presented a list of 15 words aurally and asked to freely recall these words on three occasions: (i) immediately following presentation, (ii) after a 30-minute retention interval, and (iii) after 24 hours. Crucially, the 30-minute retention interval between the immediate and 30-minute free recall tests comprised our experimental manipulation. In Session 1, participants experienced 25 minutes of higher intensity exercise (interval running) and, in Session 2, they experienced 25 minutes of lower intensity exercise (walking). Different wordlists were used in Session 1 and Session 2. The study took place in a naturalistic setting in the North East of England, UK.

A repeated measures design was employed to experimentally examine retention of aurally presented lists of words. Participants completed two study sessions spaced two weeks apart. In each session, they were presented a different list of 15 words adopted from the Rey Auditory Verbal Learning Test [RAVLT; 63] and asked to recall them freely on three occasions: (i) immediately following their presentation, (ii) after a 30-minute retention interval, and (iii) after 24 hours. Crucially, the 30-minute retention interval between the immediate and 30-minute recall tests comprised our experimental manipulation. Specifically, in Session 1, participants experienced 25 minutes of higher intensity exercise (interval running). In Session 2, they experienced 25 minutes of lower intensity exercise (walking). For practical reasons (to fit our study procedure around participants’ regular exercise training programmes), delay conditions were not counterbalanced: all participants experienced the higher intensity condition first and lower intensity condition second. The study was delivered in a naturalistic setting in North East England, UK, except for the 24-hour delayed recall tests, which were delivered remotely over the phone. Data were collected between October-December 2022. See Fig 1 for an overview of the study procedure.

### Materials

Two wordlists adopted from the RAVLT [63] were used to probe memory retention. Each wordlist contained 15 short, concrete, unrelated English nouns. Because the RAVLT has three standardised lists that are comparable in how memorable they are [e.g., 64], a different wordlist was presented in each of our two study sessions. Specifically, List A was presented in the first session and List B was presented in the second session. Verbal instructions were presented by the experimenter throughout the procedure using a set script. Responses during encoding and testing phases were collected from participants aurally and the experimenter wrote down participants’ responses. Subjective ratings of physical activity exertion were collected during the physical activity delay conditions through the Rate of Perceived Exertion scale [RPE; 62]. Objective data surrounding physical activity exertion were collected via heart rate data using participants’ own fitness watch devices (subset of participants, n = 18), and they reported these data to the researchers after the completion of the study. In addition to memory performance and physical exertion, participants’ self-reported fitness level, as well as pre- and post-study alertness, mood, and arousal were recorded. Methodological details and data analyses for these measures are reported in full in the Supplementary Information.

### Procedure

Prior to the experimental procedure, participants provided their informed consent in writing after reading a study information sheet. They were then asked to complete a short online survey that requested details surrounding their age, gender, education level, perceived fitness level, and regularity of exercise. The subsequent experimental procedure was delivered in the field. Specifically, it was completed on a seafront promenade in the North East of England, UK, where participants would typically complete exercise activities as part of their regular training programme. This was done to promote ecological validity and ensure the delay condition activities (higher intensity vs. lower intensity exercise) were in keeping with participants’ natural sporting activities and routines.

In keeping with previous work [e.g., 65], a washout period separated study sessions, which took place two weeks apart. The same experimental procedure was used across both sessions except for (i) the list of words that were encoded and recalled by participants and (ii) the activities of the 30-minute retention interval, were our experimental manipulation occurred. List A was always presented during the first study session and List B during the second study session. A higher intensity (vigorous) exercise condition was always presented during the first study session and a lower intensity (light) exercise condition was always presented during the second session.

On arrival at a study session, the researcher welcomed the participant and explained the RPE scale measure [62]. While participants (amateur endurance runners) were familiar with this measure through their regular exercise training programmes, this explanation was provided at the start of a session to minimise possible errors in applying and reporting RPE scores during the study procedure (see later). Following this, the research presented the participant with a list of 15 words from the RAVLT [63]. Words were presented aurally by the researcher at a pace of one word per second. Immediately following this, participants were asked to freely recall as many of the words as possible. There was no time limit on the time they had to recall the words. Following the immediate recall test, participants completed one of two 30-minute exercise conditions. Both conditions were completed in the same naturalistic environment to minimise the likelihood of between-session extraneous variables influencing memory performance. To ensure integrity of both conditions, the exercise activities that participants completed were designed by a qualified Triathlon Coach.

In the higher intensity exercise condition, participants were required to complete a 25-minute interval run. Specifically, they ran continuously for 12 minutes, took a 1-minute walked recovery, then ran 12 minutes back to the start point. The pace during the two 12-minute runs was alternated using equidistant lampposts placed along the route to signal interval changes: two lampposts at a fast pace followed by two lampposts at a moderate pace, repeated for the duration of the activity. Using the RPE scale as a guide, participants were asked to ensure their fast pace mirrored an RPE of 6–7 (vigorous activity) and their moderate pace mirrored an RPE of 4–5 (moderate activity). This meant that lampposts served a dual purpose: (i) to alternate physical activity intensity in keeping with typical training programmes, and (ii) to provide a cognitive task to deter the likelihood of active mnemonic strategies. The latter is especially relevant to studies examining awake consolidation, where, for example, internal rehearsal of encoded materials may reduce or negate any effect of our experimental manipulation. For the 1-minute recovery walk in the middle of this higher intensity exercise condition, participants were requested to maintain a comfortable walking pace that corresponded to an RPE of 2–3 (light activity).

In the lower intensity exercise condition, participants walked along the same route as the higher intensity condition. Specifically, they walked in one direction for 12.5 minutes before retracing their steps to provide a total duration of 25 minutes. Throughout this exercise condition, they were requested to maintain a comfortable walking pace that corresponded to an RPE of 2–3 (light activity). While physical activity intensity was experimentally manipulated between conditions, to encourage comparable mental activity in both conditions, participants were asked to count lampposts as they walked.

Following completion of the exercise condition, participants completed a 30-minute delayed free recall test for the words encoded prior to the delay. They were then asked about their experiences during the exercise condition. Specifically, they were asked to reflect on their mental activities during the delay condition to consider if any thoughts may have influenced retention of the words. Participants were asked to rate on a 5-point Likert scale how much they had (i) thought about the learned material, (ii) imagined the learned material, and (iii) tried to remember the learned material. For each measure, 1 = not at all and 5 = constant [66].

Participants were then asked to report their level of physical exertion from three times points during the delay condition: 2, 12.5 and 25 minutes from commencing the physical activity. These time points broadly reflected the start, middle, and end of the physical activity delay condition. To do this, they rated their exertion using the 10-point RPE Scale [62], where 1 corresponds to no exertion at all and 10 refers to maximal exertion. They provided a rating for each of the three noted timepoints. Heart rate data were also collected during the physical activity delay condition from a subset of participants (n = 18) using their own devices, e.g., fitness watch. Following completion of the study session, participants were asked to report the mean absolute heart rate achieved during the delay condition to the researcher. These data were used as absolute values and also converted into relative heart rate maximum (%HR max) values to control for age [67]. In addition to heart rate values, participants reported their RPE scores.

Twenty-four hours after a study session, participants completed a 24-hour delayed recall test over the phone, where they were again asked to freely recall the items from the wordlist encoded one day earlier. To encourage the ecological validity of our methods and outcomes, we did not control participants activities (e.g., sleep, diet, or physical activity) during the interval between the 30-minute and 24-hour recall tests. Participants were free to go about their normal activities between testing sessions, which is not uncommon in paradigms investigating the effect of post-encoding activities on early consolidation [16, 68]. Like for the 30-minute test, participants were not informed in advance that they would complete a further free recall test for the wordlist, but they were aware that the researchers would follow up with them at this time. This was done to reduce the likelihood of mnemonic strategies between the 30-minute and 24-hour delayed recall tests though participants will have naturally expected a 30-minute and 24-hour recall test for the words encoded in study session two due to having already experienced the same protocol during the first study session.

### Scoring

To examine the retention of wordlist materials, the total number of words recalled correctly was extracted for the (i) immediate, (ii) 30-minute, and (iii) 24-hour recall tests. To control for individual differences in immediate recall and between-condition variation, percentage retention scores were also computed for the 30-minute and 24-hour delayed recall tests to establish how many words recalled at the immediate stage were retained over the duration of these retention intervals [16, 25, 69]. These percentage retention scores were calculated by dividing the total number of words recalled correctly in the 30-minute and 24-hour tests by the total number of words recalled correctly in the immediate test. These values were then multiplied by 100 to provide a percentage score. In keeping with previous consolidation research [e.g., 22], scores were capped at 100%. If the participant recalled a word in the 30-minute or 24-hour test that was not recalled in a previous recall test, it was still recorded as correct. Mean heart rate data were collected from a subset of participants (n = 18) and converted to %HRmax scores [67] using the formula: %HRmax = (Heart Rate / Maximum Heart Rate) * 100, where Maximum Heart Rate was computed as 220 minus a participant’s age, e.g., 220–40 = 180 Maximum Heart Rate. Post-experimental ratings regarding thoughts about encoded materials were scored in keeping with published work [12], where raw scores were extracted for analyses, and this was also the case for the RPE Scale [62].

### Statistical analyses

Analyses were performed using SPSS Statistics 28 (copyright IBM Corp., NY, USA), with the alpha level set to .05. Raw memory scores (i.e., number of words recalled) were analysed using a 3x2 repeated measures ANOVA that used within-subject factors time of test (3 levels: immediate vs. 30-minute vs. 24-hour) and exercise intensity condition (2 levels: higher vs. lower). Further to this, to control for individual differences in encoding and immediate recall, percentage retention scores for the 30-minute and 24-hour tests were analysed using a 2x2 ANOVA that used within subject factors time of test (2 levels: 30-minutes vs. 24-hour) and exercise intensity condition (2 levels: higher vs. lower). Paired t-tests (two tailed) were used to examine possible differences in raw and percentage retention scores between conditions or time points, for example, comparison of raw scores between conditions in the immediate, 30-minute, and 24-hour recall tests. Data from the post-experimental questionnaire (e.g., ratings surrounding the degree to which participants thought about the wordlists during the exercise delay conditions) were compared between conditions using a paired t-test (two tailed). Mean heart rate and %HR max scores were also compared between conditions using paired t-tests (two tailed). Finally, RPE scores were examined using a 3x2 repeated measures ANOVA that used within-subject factors time of test (3 levels: 2 minutes vs. 12.5 minutes vs. 25 minutes) and exercise intensity condition (2 levels: higher vs. lower), as well as paired t-tests to examine possible differences in RPE scores between conditions at the three time points. In all cases where multiple paired t-tests were conducted, Bonferroni-corrected alpha levels (p = .05 / number of within-family comparisons) were used to reduce the likelihood of Type I errors. All study data are available on the project OSF site at osf.io/xds49.

## Results

### Memory performance

Table 1 reports the mean number of words recalled in the immediate, 30-minute, and 24-hour recall tests for the higher intensity and lower intensity conditions. A 3x2 repeated measures ANOVA revealed significant main effects of exercise condition (F(1,34) = 8.977, p = .005, η_p_^2^ = .209) and time of test (F(2,68) = 118.454, p < .001, η_p_^2^ = .777), as well as a significant interaction between exercise condition and time of test (F(2,68) = 6.826, p = .002, η_p_^2^ = .167). Paired t-tests (two-tailed) demonstrated that the number of words recalled in the immediate test did not differ significantly between the higher intensity and lower intensity exercise conditions (t(34) = 0.848, p = .402, d = 0.143), but the number of words recalled in the higher intensity condition was significantly greater when memory was probed in the 30-minute recall test (t(34) = 3.686, p < .001, d = 0.623) and 24-hour recall test (t(34) = 3.954, p < .001, d = 0.668). These significant findings survived Bonferroni-corrected alpha levels of p = .017 (p = .050 / 3 comparisons) that controlled for multiple within-family comparisons.

**Table 1:** Recall performance.

Test time	Lower intensity	Higher intensity	t-test (two tailed)
Immediate test	8.06 (2.28)	8.37 (1.65)	t(34) = 0.848, p = .402, d = 0.143
30-minute test	5.66 (2.31)	6.71 (1.82)	t(34) = 3.686, p < .001, d = 0.623
24-hour test	4.97 (2.15)	6.17 (1.89)	t(34) = 3.954, p < .001, d = 0.668

The mean number of words recalled in the immediate, 30-minute, and 24-hour recall tests are shown for the higher intensity and lower intensity conditions. Standard deviation values are reported in parentheses. Outcomes from paired t-tests (two-tailed) are also reported. The number of words recalled in the immediate recall test was comparable between conditions, but participants recalled significantly more words in the 30-minute and 24-hour recall tests in the higher intensity exercise condition.

Meanwhile, comparison of data between testing timepoints revealed that participants recalled significantly fewer words in the 30-minute recall test than immediate recall test (lower intensity: (t(34) = 8.806, p < .001, d = 1.488; higher intensity: t(34) = 8.261, p < .001, d = 1.396), and significantly fewer words in the 24-hour recall test than 30-minute recall test (lower intensity: t(34) = 4.680, p < .001, d = 0.791; higher intensity: t(34) = 4.117, p < .001, d = 0.696), i.e., forgetting of wordlist materials occurred between each of the testing points. These significant findings survived a Bonferroni-corrected alpha level of p = .013 (p = .050 / 4 comparisons) that controlled for multiple within-family comparisons.

Taken together, these outcomes demonstrate that participants experienced significant forgetting of wordlist material between testing time points in both conditions, but especially in the lower intensity exercise condition. This is reflected in Table 1, where a mean of 2.20 words were forgotten between the immediate recall test and 24-hour recall test in the higher intensity condition, whereas a mean of 3.09 words were forgotten between the immediate and 24-hour recall tests in the lower intensity condition.

**Fig 2:**
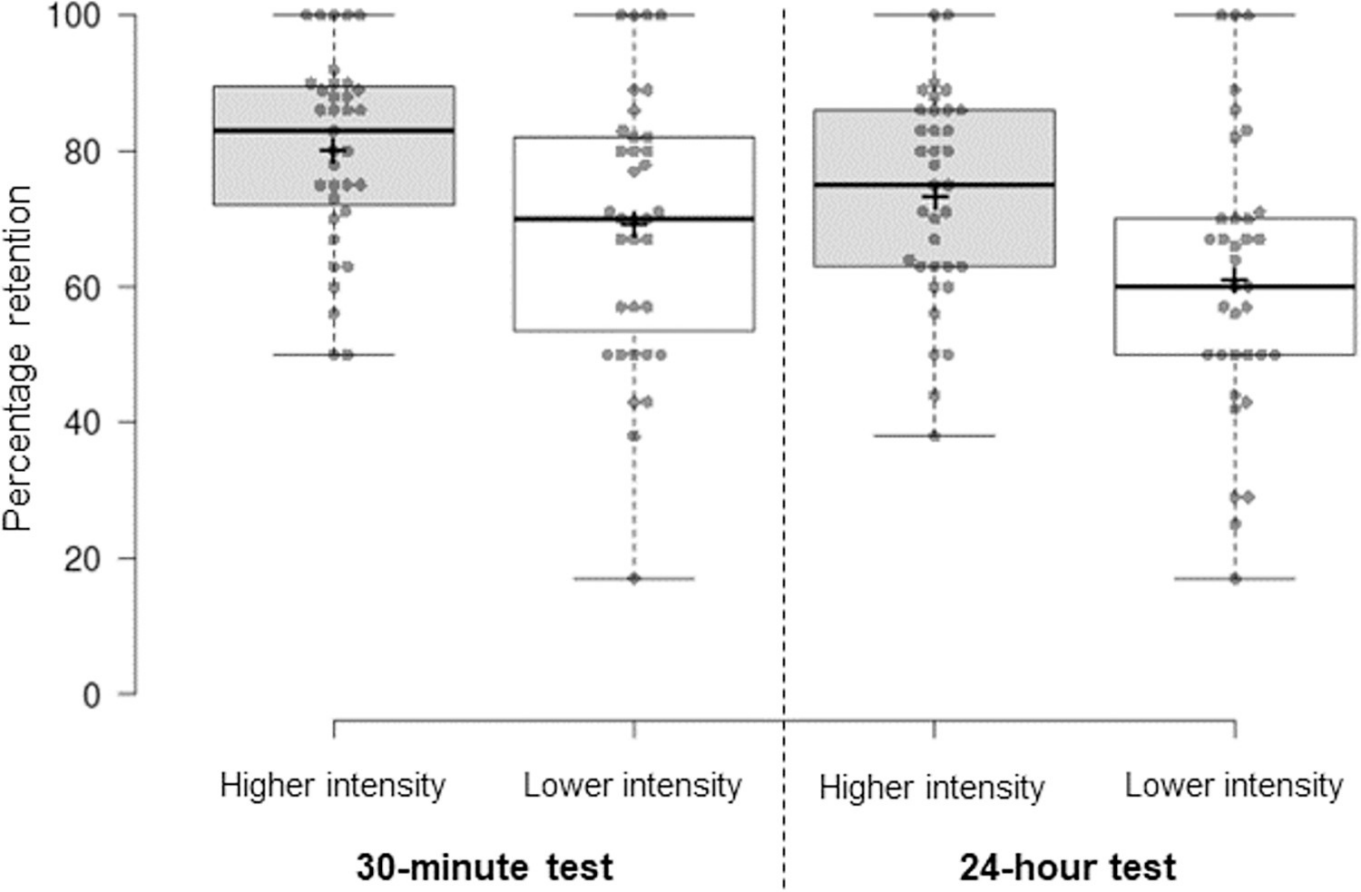
Memory retention scores. The box plot shows percentage retention scores for the higher intensity (grey boxes) and lower intensity (white boxes) exercise conditions from the 30-minute and 24-hour test. Percentage retention scores were computed as the number of words recalled correctly in a delayed test divided by the number of words recalled correctly in the immediate test, multiplied by 100. Centre lines show the medians; box limits indicate the 25th and 75th percentiles; whiskers extend to minimum and maximum values; crosses represent sample means; data points are plotted as open circles. Percentage retention scores were significantly greater in the higher intensity than lower intensity condition in the 30-minute and this effect was maintained in the 24-hour test.

Further to examining raw recall scores, percentage retention scores, which account for individual differences in the encoding of presented materials, were analysed. Percentage retention scores for the 30-minute and 24-hour recall tests are provided in Fig 2. A 2x2 repeated measures ANOVA revealed that wordlist retention was significantly greater in the higher intensity condition than in the lower intensity condition (F(1,34) = 14.763, p < .001, η_p_^2^ = .303). A significant main effect of time was also observed because retention of words was poorer when memory was probed after 24 hours than 30 minutes (F(1,34) = 34.861, p < .001, η_p_^2^ = .506), but no significant interaction was observed between delay condition and time of test (F(1,34) = 0.445, p = .509, η_p_^2^ = .013). This indicates that, when controlling for individual differences in encoding, the rate of forgetting between the 30-minute and 24-hour recall tests was comparable in both conditions. Paired t-tests confirmed that percentage retention scores were significantly greater in the higher intensity than lower intensity condition in the 30-minute delayed recall test (t(34) = 3.641, p < .001, d = .615) and 24-hour delayed recall test (t(34) = 3.635, p < .001, d = .614). Similarly, they confirmed that there was a significant decline in percentage retention scores between the 30-minute and 24-hour recall tests in both the higher intensity (t(34) = 4.193, p < .001, d = .709) and lower intensity (t(34) = 4.842, p < .001, d = .818) exercise conditions. These significant findings survived Bonferroni-corrected alpha levels of p = .025 (p = .050 / 2 comparisons) to correct for multiple within-family comparisons.

### Post-experimental questionnaire

Following completion of the 30-minute delayed recall test, participants were asked about their experiences during the exercise condition. Data from one participant was lost for this measure and thus the following analyses are reported from a sample of n = 34. Participants responses for this measure comprised rating, on a 5-point Likert scale, how often they (i) thought about (higher intensity: M = 1.41, SD = 0.74; lower intensity: M = 1.74, SD = 1.02; t(33) = -1.683, p = .102, d = -.289), (ii) imagined (higher intensity: M = 1.35, SD = 0.54; lower intensity: M = 1.62, SD = 0.99; t(33) = -1.358, p = .184, d = -.233), or (iii) tried to remember (higher intensity: M = 1.47, SD = 0.83; lower intensity: M = 1.56, SD = 0.99; t(33) = -0.415, p = .681, d = -.071) the wordlist materials, where higher scores reflect greater activity [12]. Scores across all three dimensions were comparable and relatively low, indicating that (i) participants did not actively engage in mnemonic strategies during the study and (ii) the level of mental activity surrounding the wordlists was comparable in the lower intensity and higher intensity exercise conditions.

### Physical exertion

#### Heart rate

To quantify the level of physiological exertion experienced by participants, heart rate data were recorded from a subset of individuals (n = 18) throughout the higher intensity and lower intensity exercise delay conditions. A paired t-test confirmed that mean absolute heart rate values were significantly greater (t(17) = 18.118, p < .001, d = 4.270) in the higher intensity condition (M = 154.44, SD = 11.63) than in the lower intensity condition (M = 87.00; SD = 12.70). Similarly, when relative heart rate maximum (%HRmax) values were computed, participants demonstrated significantly greater %HRmax scores (t(17) = -19.766, p < .001, d = -4.659) in the higher intensity condition (M = 89.27, SD = 5.08) than the lower intensity condition (M = 50.39, SD = 7.75). Mean %HRmax values in the lower and higher intensity conditions aligned with published thresholds [70] for *light intensity* exercise (%HRmax score of 40 < 55) and *vigorous intensity* exercise (%HRmax score of 70 < 90), respectively. Both findings survived a Bonferroni-corrected alpha level of .025 (p = .050 / 2 comparisons) to correct for multiple within-family comparisons.

Pearson correlations provided no evidence for a relationship between mean absolute heart rate values and memory performance (both conditions p > .122). This was also true for %HRmax values and memory performance (both conditions p > .326).

### Rate of Perceived Exertion (RPE)

To complement objective heart rate data, participants were asked to provide subjective reports of their RPE [62]. Table 2 shows mean scores for both conditions for measurements taken 2, 12.5, and 25 minutes after commencing the two exercise conditions. Higher intensity condition responses were indicative of *moderate to vigorous* physical exertion, whereas lower intensity condition responses were indicative of *light* physical exertion. These data, combined with the heart rate data reported earlier, indicate that participants exercised at the desired intensity in our two delay conditions.

**Table 2:** Rate of perceived exertion (RPE).

Measurement time	Condition	RPE score	Range	Descriptor
2 minutes	Higher intensity	5.31 (1.71)	2–8	Moderate activity
	Lower intensity	2.29 (1.49)	1–8	Light activity
12.5 minutes	Higher intensity	6.54 (1.04)	4–8	Vigorous activity
	Lower intensity	2.46 (1.38)	1–7	Light activity
25 minutes	Higher intensity	7.77 (1.35)	4–10	Vigorous activity
	Lower intensity	2.51 (1.34)	1–7	Light activity

Mean RPE scores, standard deviations, and the range of scores for the higher intensity and lower intensity exercise conditions are shown for measurements collected 2, 12.5, and 25 minutes after commencing physical activity. Category descriptors for the mean scores are also shown.

A 3x2 repeated measures ANOVA revealed a significant main effect of exercise condition, where, expectedly, higher exertion scores were reported in the higher intensity condition (F(1,34) = 318.317, p < .001, η_p_^2^ = .903). A significant main effect of time was also observed, where participants reported greater exertion as the physical activity progressed (F(2,68) = 26.341, p < .001, η_p_^2^ = .437). The interaction between exercise condition and time was found to be significant (F(2,68) = 22.462, p < .001, η_p_^2^ = .398) because a greater increase in exertion levels over time was observed in the higher intensity than lower intensity condition, where scores for the latter remained relative stable across the delay (see Table 2). Paired t-tests (two tailed) confirmed that the difference between conditions was significant at each time point (all p < .001). The increase in exertion levels over time in the higher intensity condition was significant between measurements taken at 2 minutes and 12.5 minutes (t(35) = -3.605, p < .001, d = -.609) and 12.5 minutes and 25 minutes (t(34) = -5.375, p < .001, d = -.909). In the lower intensity condition, no significant change in scores was observed between measurements taken at 2 minute and 12.5 minutes (t(34) = -1.974, p = .057, d = -.334) or those taken at 12.5 and 25 minutes (t(34) = -1.435, p = .160, d = -.243). Significant findings survived a Bonferroni-corrected alpha level of p = 0.125 (p = 0.05 / 4 comparisons) that controlled for multiple within-family comparisons.

## Discussion

The purpose of the current study was to examine whether lower and higher intensity exercise completed in a naturalistic setting differentially affect the early consolidation of new declarative memories. To achieve this, we adapted an established consolidation paradigm used in laboratory research [10, 15, 16] to accommodate the normal exercise activities of adult endurance runners. Wordlist retention probed 30 minutes and 24 hours after encoding was significantly better when participants experienced higher intensity (interval running) exercise than lower intensity (walking) exercise. Our finding of superior memory following higher intensity exercise reinforces existing laboratory work [28, 32, 33, 35, 71] and, crucially, demonstrates that such effects translate to naturalistic settings. We consider possible explanations for our findings and the further work that is required for applied consequences to be achieved, for example, in educational environments and clinical populations.

Why did higher intensity exercise benefit the retention of new memories? Our repeated measures design allows us to rule out the contribution of random noise between participants that could affect an independent samples design. The reported effect is also unlikely to be explained by within-subject differences in participants’ mental state and mood between sessions: comparable alertness, arousal, and mood levels were reported prior to completing study sessions (see SI). Similarly, the effect in retention is unlikely to be explained by variations in the initial encoding of wordlist materials: participants received the same treatment during the encoding of both wordlists and immediate memory performance was matched across the two conditions. This is pertinent given that different wordlists from the RAVLT [63] were presented in our two study sessions, though current evidence highlights that the employed lists (A and B) are comparably memorable [e.g., 64]. Given that a difference in memory retention only emerged following the 30-minute retention interval, it is most likely that activities that occurred during the retention interval–where our experimental manipulation occurred–were responsible for the observed differences in delayed memory performance.

It is possible that the reported effect may simply be a direct result of mnemonic strategies (e.g., intentional rehearsal) during the 30-minute retention interval between the immediate and 30-minute recall tests. However, this appears unlikely. In keeping with existing consolidation research [10, 16, 69], we attempted to minimise the likelihood of participants engaging in mnemonic strategies by (i) embedding a cognitive task (lamppost counting) during both conditions and (ii) not informing participants that they would complete subsequent (30-minute and 24-hour) recall tests for the encoded materials. Of course, given that our sessions were not counterbalanced for practical purposes (to fit our study procedure around participants’ existing exercise training programmes), participants will have been aware that their memory for the wordlist will be tested at multiple time points in the second study session. If this knowledge affected our study outcomes, for example, because participants actively rehearsed materials in the second session, superior retention would be expected in the second (walk) than first (run) session. Conversely, participants demonstrated superior delayed memory for materials in the *first* session. This is reflected in our data, which demonstrate that participants reported thinking about, imagining, and remembering the wordlists to comparable level in the two conditions. Thus, differences in (conscious) mental activities during the delay conditions are unlikely to account for the reported effect in memory. Furthermore, by ensuring the duration of the conditions was equivalent, the most plausible explanation for the differences observed in memory retention is the experimental manipulation of exercise intensity.

We propose that the most likely explanation for our findings is that higher intensity exercise benefited memory retention, relative to lower intensity exercise, because it provided a state that was more conducive to the early consolidation of new wordlist memories. This consolidation hypothesis is in keeping with existing laboratory findings from work in humans and rodents [28, 32, 33, 35, 71]. At a neurobiological level, it is plausible that the higher intensity condition resulted in greater blood flow, including to brain regions implicated in memory functioning, namely the hippocampus and associated cortical networks [1, 3, 18]. Indeed, heart rate and RPE data demonstrated that participants (i) experienced a significantly greater level of exertion in the higher intensity condition, and (ii) the rate of exertion in the higher intensity condition and lower intensity condition corresponded to vigorous and light exertion, respectively, according to published thresholds [70].

It is therefore possible that participants experienced greater brain blood flow and oxygen availability in the higher intensity condition, which encouraged neurobiological mechanisms of consolidation. Indeed, even a brief period of moderate exercise can increase hippocampal blood flow [46, 47]. This may have also been coupled with increased concentrations of neurotransmitters including BNDF [48, 49] and the induction of LTP and synaptic plasticity [50], which are implicated in the success of consolidation [51–53]. It also remains possible that a physiological stress response, through activation of the Hypothalamic-Pituitary-Adrenal (HPA) axis and release of cortisol, facilitated consolidation in the higher intensity exercise condition and not the lower intensity exercise condition. This possibility resonates with (i) the well-documented U-shaped dose-response relationship between stress and cortisol in the hippocampus [42], where cortisol concentrations predicts consolidation functioning [72], and (ii) data from the current study showing that RPE and %HRmax scores in the higher intensity group corresponded to a state of vigorous exercise, which may have induced a stress response that was sufficient to encourage consolidation but not excessive to the point of inducing detrimental effects on memory [73]. It is, however, worth noting that our assessment of exercise intensity comes with some limitations. Heart rate data, which were our primary physiological indicator of exercise intensity, were only collected from a subset of participants, and not the full sample. Furthermore, these data were collected from participants’ own fitness devices, which could have influenced the accuracy of data and contributed to the between and within-subject variability. Still, when considered in its entirety and alongside RPE scores, our data do suggest that the two exercise conditions differed in their intensity level. Therefore, it is possible that a neurobiological explanation could, at least partly, explain the observed difference in memory retention between our higher intensity and lower intensity exercise conditions.

In addition to a neurophysiological explanation, we cannot rule out the possibility that–above and beyond providing a conducive neurophysiological state for consolidation–higher intensity exercise may have evoked a more efficient cognitive state of arousal and improved participants’ mood, which facilitated the recall of information. While less likely for the 24-hour recall test that was completed after a washout period, this possibility is pertinent to the 30-minute recall test, which occurred in close proximity to the exercise condition. Our data do resonate with this possibility: while significant improvements in alertness, mood, and arousal were observed following both exercise conditions, there improvements were greater when the exercise condition was higher in intensity (see S1 Appendix). It is well established that human cognitive processes, including learning and memory, can be influenced by affective states [74, 75]. Indeed, it is proposed that exercise has a modulating effect on memory and information is better remembered when paired with an emotional stimulus, such as high intensity exercise [76]. It is evident from our data that higher levels of alertness, arousal and a more positive mood state followed completion of the high-intensity physical activity condition. Thus, further work is required to understand the possible contribution of affective states to our findings and to exclude that the reported effect in memory retention can be explained by enhanced retrieval without enhancement of consolidation. Still, the latter appears less likely given that (i) the benefit of higher intensity exercise remained after 24 hours when improvements in alertness, mood, and arousal would have been expected to have dissipated by this point, and (ii) existing work demonstrates that acute, vigorous exercise is conducive to consolidation and memory retention when controlling for possible effects in retrieval, e.g., through probing memory after an extended delay [36].

We also cannot rule out the possibility that other phenomenon, for example, behavioural tagging [77], where the consolidation of malleable memory traces can be enhanced through post-encoding novelty [78–80] may have contributed to our findings. Such novelty is proposed to support consolidation through stimulating the release of neurotransmitters including dopamine and the encouragement of protein synthesis in the hippocampus [81]. Indeed, navigation of virtual environments has been shown to be sufficient to induce such novelty [78, 79] though findings are mixed [82]. In the current study, it is possible that higher intensity physical activity in a natural setting provided such novelty in the first condition, whereas walking in the same environment for the lower intensity condition two weeks later did not induce such novelty. While this remains possible, it is less likely given that participants were already highly familiar with the natural study environment because they train in this environment on a regular basis. Indeed, to encourage the ecological validity of our study, our measures were built around their typical training activities, including the interval run in the higher intensity condition. Still, further work is required to clarify the possible cognitive, affective, and neurophysiological bases of the reported effect in memory retention.

Irrespective of the mechanisms that underpin the observed effect, it is noteworthy that it was not transient but long lasting and detectable after at least 24 hours. This demonstrates that the effect of acute exercise in naturalistic settings is durable and can be observed even after an overnight period of sleep, which is in keeping with laboratory-based work [34, 35, 40]. Crucial to the aims of the current study, we observed this durable effect in memory retention even when our procedure was delivered in a naturalistic setting and when not controlling for participants’ activities (e.g., sleep, physical activity) in the interval between the 30-minute and 24-hour test. To promote ecological validity, our study procedure, which was based on published laboratory work [10, 15, 16], was moulded around participants normal exercise activities. It was for this reason that we recruited endurance runners for this initial investigation. We acknowledge that this does however limit the generalisability of our findings and further work is required to establish whether the characteristics of recruited individuals influenced outcomes. In particular, it would be valuable to identify whether the longer-term (24-hour) effect in the current study is observable in those who do not practice exercise regularly given that chronic exercise is proposed to encourage the molecular framework for consolidation to succeed [26]. Thus, it is possible that our sample of chronic exercisers already existed in a neurophysiological state that was conducive to consolidation.

Rigorous standardisation procedures were applied in the current study to reduce the likelihood of any potential contextual and extraneous differences that may have influenced the outcome of the study and/or introduced bias. The importance of mitigating bias when comparing exercise modes in terms setting and load is deemed important in exercise research [83]. Despite our efforts to address both of those elements, we set out to examine the effects of exercise on memory in a natural setting, to reflect a real-life scenario. Consequently, there was a higher risk of compromise from ecological factors, for example, differences in temperature and weather conditions, that are easier to address in a controlled laboratory-based setting. This includes likely inter-individual differences in heart rate data due to participants using their own devices [84, 85]. However, the specific aim of this study was to observe an effect of exercise in a natural setting because many people choose to carry out exercise in parks, fields, streets, towns, countryside, and coastlines and in all weather conditions. For these reasons, we chose to conduct the experiment in the field so we could be confident that the results would be more generalisable to a ‘real life’ scenario. Despite the scientific limitations that this approach brings, we achieved this aim.

Can our findings have applied consequences? While it is generally accepted that exercise has significant benefits for physical and mental well-being throughout the lifespan [58], to the best of our knowledge, our data provide the first field-based evidence that an acute bout of higher intensity exercise positively affects the retention of new declarative memories, relative to lower intensity exercise. As a result, it is possible that our findings may provide tentative evidence for interventions or lifestyle changes that abate forgetting. However, for this to be achievable, several questions must be addressed. For example, what are the precise physiological (and cognitive) mechanisms that underly the observed effect and what level of physical activity is sufficient–and optimal—to achieve the effect across individuals of varying abilities consistently? Exploring related questions in children and older adults with intact and comprised memory systems may also provide valuable insights. Children who practice regular aerobic activity have been found to perform better in verbal, perceptual and arithmetic tests [86, 87]. Similarly, contemporary evidence indicates that acute and chronic physical activity in older age is associated positively with episodic memory [88, 89]. If the reported effect is translatable across different settings and populations and the underlying neurophysiological mechanisms can be established, this could lead to novel targets for interventions and memory-enhancing recommendations that can be applied, for example, in education and care settings. Furthermore, given that findings–including our own–demonstrate that exercise not only benefits cognition but can have broader effects, for example, in arousal and mood, there may be scope for interventions in clinical populations, including those with depression who often report reduced mood and memory functioning [60, 90, 91]. If future work can characterise the criteria required to achieve a memory benefit of acute exercise in naturalistic settings, this can provide a significant stepping stone towards healthcare professionals’ ability to recommend (at least) short bouts of higher intensity exercise as a non-invasive memory-enhancing intervention.

## Conclusions

Our data indicate that higher intensity exercise in the minutes immediately following new learning benefited the long-term retention of new verbal memories relative to a period of lower intensity exercise. Crucially, this finding was observed in the field and provides evidence that laboratory-based findings demonstrating that acute exercise benefits memory retention translates to naturalistic settings. We attribute this finding to a consolidation account, where higher intensity exercise is likely to have provided a physiological (and possibly cognitive) state that was more conducive to the early stabilisation and strengthening of new declarative memories, relative to lower intensity exercise. While further work is required to qualify the scope of this finding and its neurocognitive basis, it provides tentative evidence that exercise interventions can support the retention of new memories in naturalistic settings.

## Supporting information

S1 Appendix Additional methodological details and results. The supporting information document contains (i) self-reported fitness level scores measured using the framework of the International Fitness Scale (IFIS), (ii) pre- and post-study alertness scores measured through the Stamford Sleepiness Scale (Hoddes et al., 1973), (iii) pre- and post-study mood scores measured through the Feeling Scale (Hardy et al., 1999) before and after the 30-minute physical activity condition, and (iv) pre- and post-study arousal scores measured using the Felt Arousal Scale (Svebak & Murgatroyd, 1985).
(DOCX)
