# Supplementary material for: Higher intensity exercise after encoding is more conducive to episodic memory retention than lower intensity exercise: A field study in endurance runners
Source: PLoS One. 2024 Sep 13;19(9):e0308373. doi: 10.1371/journal.pone.0308373 (PMC11398685; doi:10.1371/journal.pone.0308373)
Supplement: S1 Appendix — The supporting information document contains (i) self-reported fitness level scores measured using the framework of the International Fitness Scale (IFIS), (ii) pre- and post-study alertness scores measured through the Stamford Sleepiness Scale (Hoddes et al., 1973), (iii) pre- and post-study mood scores measured through the Feeling Scale (Hardy et al., 1999) before and after the 30-minute physical activity condition, and (iv) pre- and post-study arousal scores measured using the Felt Arousal Scale (Svebak & Murgatroyd, 1985). (DOCX) [file pone.0308373.s001.docx]

Higher intensity exercise after encoding is more conducive to episodic memory retention than lower intensity exercise: a field study in endurance runners

Roger Makepeace^1^ & Michael Craig^1*^

^1^ Department of Psychology, Faculty of Health and Life Sciences, Northumbria University, Newcastle upon Tyne, UK

^*^ Corresponding author

Email: michael2.craig@northumbria.ac.uk (MC)

# Supplementary Methods

**Pre-study reports of sleep, exercise, and fitness levels**

In addition to the methods reported in our manuscript, prior to completing the study participants were requested to report their typical sleep duration, exercise activity, and current fitness level. Sleep duration was measured through requesting that participants report the average (mean) number of hours that they slept overnight in the week that preceded the study. Participants were then asked to note the average number of minutes that they typically engage in moderate and intense exercise in a normal week. General fitness levels were measured using the International Fitness Scale [IFIS; 1]. This scale-based measure comprises four forms of fitness: (i) cardiovascular, (ii) muscular strength, (iii) speed and agility, and (iv) flexibility. For each item, participants are requested to rate their fitness on a 5-point Likert scale from 1 (very poor) to 5 (very good), where 3 = average. Participants also provided an overall rating of the general fitness level using the same scale. Raw values for each measure were extracted for the purpose of analyses.

**Pre- and post-study reports of alertness, mood, and arousal**

Participants also provided ratings of their alertness, mood, and arousal immediately preceding and immediately after the study procedure, i.e., prior to the encoding of a list of words and after the 30-minute free recall of the words. Alertness was measured through the Stanford Sleepiness Scale [2], which required participants to rate their current level of alertness on a 7-point Likert scale, where 1 = “Feeling active, vital, alert or wide awake” and 7 = “sleepy, woozy, prefer to life down”. Mood was measured through the Feeling Scale [3]. This scale-based measure asks a user to rate their mood at the current moment in time on an 11-point Likert scale ranging from -5 (very bad) to +5 (very good). Arousal was measured using the Felt Arousal Scale [FAS: 4]. The FAS is a 6-point rating scale measuring affective responses on the arousal dimension from 1 (“low arousal”) to 6 (“high arousal”). Raw values for each measure were extracted for the purpose of analyses.

# Supplementary Results

**Pre-study reports of sleep, exercise, and fitness levels**

Participants reported an average of 7.14 hours (SD = 0.87, range = 6-9 hours) of sleep per night in the week preceding the study. When asked about their typical physical activity levels in a normal week, they reported completing an average of 223.00 minutes (SD = 107.00 minutes, range = 60-430 minutes) of moderate exercise and 106 minutes (SD = 71.40 minutes, range = 0-300 minutes) of vigorous exercise. Probing of general fitness levels using the IFIS revealed that most participants (n = 33/35, 94.30%) reported their overall fitness as good to very good (see Table S1). When subcategories were examined, participants (endurance runners) rated themselves more highly on scales of cardiovascular fitness than muscular strength, and speed and agility. Ratings of flexibility suggest that participants considered their fitness in this category as average to good.

| **Table S1. Perceived fitness.** | | | | | | |
| --- | --- | --- | --- | --- | --- | --- |
| **Area of fitness** | **Mean score (SD)** | | **Range** | | **Range descriptors** | |
| Overall fitness | 4.50 (0.60) | | 3-5 | | Good – Very good | |
| Cardiovascular | 4.34 (0.68) | | 3-5 | | Good – Very good | |
| Muscular strength | 3.60 (0.65) | | 3-5 | | Average – Very good | |
| Speed and agility | 3.78 (0.73) | | 2-5 | | Average – Very good | |
| Flexibility | 3.11 (0.72) | | 2-4 | | Average – Good | |
| Self-reported fitness levels for the sample are reported in the table and accompanied by descriptors for mean scores. Participants rated their current fitness using the framework of the International Fitness Scale (IFIS). | | | | | | |
|  |  |  | |  | |  |

**Pre- and post-study reports of alertness, mood, and arousal**

**Alertness**

Table S2 reports mean ratings where a score of 1 is the highest level of alertness and 7 is the lowest. Scores indicate that participants were functioning at high but not peak levels of alertness before engaging in the exercise conditions. A 2x2 repeated measures ANOVA using within-subject factors exercise condition (2 levels: lower vs. higher) and time of test (2 levels: pre-condition vs. post-condition) revealed no significant main effect of condition (F(1,34) = 0.849, p = .364, η_p_^2^ = .025), but a significant main effect of time was observed (F(1,34) = 36.913, p < .001, η_p_^2^ = .528) because participants reported feeling more alert after than before the exercise condition. A significant interaction between condition and time was also found (F(1,34) = 5.408, p = .026, η_p_^2^ = .141) because the reduction in alertness was more pronounced in the higher intensity condition than the lower intensity condition. Paired t-tests (two tailed) revealed that alertness ratings were comparable prior to the exercise condition (t(34) = 0.000, p = .500, d = 0.000) but differed significantly following the exercise condition (t(34) = -2.098, p = .022, d = -0.360). For both conditions, participants reported feeling more alert (i.e., more alert) after the exercise condition. This change was significant in both the high-intensity (run) condition (t(34) = 6.263, p < .001, d = 1.059) and low-intensity (walk) condition (t(33) = -2.925, p = .003, d = -.502). All significant findings survived Bonferroni-corrected alpha levels of p = .025 (p = .050 / 2 comparisons) to correct for multiple within-family comparisons.

| **Table S2. Alertness scores.** | | |
| --- | --- | --- |
| **Measurement time** | **Condition** | **Mean score (SD)** |
| Pre-condition | Higher intensity | 2.31 (0.7) |
|  | Lower intensity | 2.31 (0.96) |
| Post-condition | Higher intensity | 1.47 (0.66) |
|  | Lower intensity | 1.82 (0.67) |
| Average alertness scores as measured through the Stamford Sleepiness Scale (Hoddes et al., 1973) before and after the 30-minute physical activity condition. | | |

**Mood**

Table S3 shows mean mood ratings from before and after the two delay conditions, as measured using the Feeling Scale [3]. A 2x2 repeated measures ANOVA using within-subject factors exercise condition (2 levels: lower vs. higher) and time of test (2 levels: pre-condition vs. post-condition) revealed no significant main effect of condition (F(1,34) = 1.479, p = .232, η_p_^2^ = .042), but a significant main effect of time was observed (F(1,34) = 44.133, p < .001, η_p_^2^ = .042) because participants reported feeling more positive after than before the exercise condition. A significant interaction between condition and time was also found (F(1,34) = 9.759, p = .004, η_p_^2^ = .223) because the improvement in mood was more pronounced in the higher intensity condition than the lower intensity condition. Paired t-tests (two tailed) revealed that ratings of mood were comparable prior to the exercise condition (t(34) = -1.165, p = .126, d = -0.197) but differed significantly following the exercise condition (t(34) = 2.163, p = .019, d = 0.021). For both conditions, participants reported a more positive mood after both delay conditions. These positive changes in mood were significant in both the high-intensity (run) condition (t(34) = -6.332, p < .001, d = -1.070) and low-intensity (walk) condition (t(34) = -3.639, p < .001, d = -.615). All significant findings survived Bonferroni-corrected alpha levels of p = .025 (p = .050 / 2 comparisons) to correct for multiple within-family comparisons.

| **Table S3. Mood scores.** | | |
| --- | --- | --- |
| **Measurement time** | **Condition** | **Mean score (SD)** |
| Pre-condition | Higher intensity | 1.83 (2.02) |
|  | Lower intensity | 2.69 (1.80) |
| Post-condition | Higher intensity | 3.97 (0.89) |
|  | Lower intensity | 3.63 (1.03) |
| Mean mood scores as measured through the Feeling Scale (Hardy et al., 1999) before and after the 30-minute physical activity condition. | | |

**Arousal**

Table S4 shows the mean scores for arousal ratings measured before and after the delay conditions using the Felt Arousal Scale [FAS: 4]. A 2x2 repeated measures ANOVA using within-subject factors exercise condition (2 levels: lower vs. higher) and time of test (2 levels: pre-condition vs. post-condition) revealed no significant main effect of condition (F(1,34) = 0.062, p = .805, η_p_^2^ = .002), but a significant main effect of time was observed (F(1,34) = 41.470, p < .001, η_p_^2^ = .549) because participants reported greater arousal after than before the exercise condition. A significant interaction between condition and time was also found (F(1,34) = 14.340, p < .001, η_p_^2^ = .297) because the improvement in arousal was more pronounced in the higher intensity condition than the lower intensity condition. Paired t-tests (two tailed) revealed that ratings of arousal were comparable prior to the exercise condition (t(34) = -1.575, p = .062, d = -0.266) but differed significantly following the exercise condition (t(34) = 2.758, p = .005, d = 0.466). Significant improvements in arousal were observed in both delay conditions (run: t(34) = -6.215, p < .001, d = -1.070; walk: t(34) = -3.260, p = .003, d = -.551). The reported mean scores across delay conditions and testing timepoints are reflective of moderate to good levels of arousal. All significant findings survived Bonferroni-corrected alpha levels of p = .025 (p = .050 / 2 comparisons) to correct for multiple within-family comparisons.

| **Table S4. Arousal scores.** | | |
| --- | --- | --- |
| **Measurement time** | **Condition** | **Mean score (SD)** |
| Pre-condition | Higher intensity | 3.60 (1.24) |
|  | Lower intensity | 3.97 (1.07) |
| Post-condition | Higher intensity | 4.86 (0.97) |
|  | Lower intensity | 4.40 (0.70) |
| Mean scores on the Felt Arousal Scale (Svebak & Murgatroyd, 1985) before and after the 30-minute physical activity condition. | | |

**References**

1. Ortega FB, Ruiz JR, España-Romero V, Vicente-Rodriguez G, Martínez-Gómez D, Manios Y, et al. The International Fitness Scale (IFIS): usefulness of self-reported fitness in youth. Int J Epidemiol. 2011;40(3):701-11. Epub 2011/03/29. doi: 10.1093/ije/dyr039. PubMed PMID: 21441238.

2. Hoddes E, Zarcone V, Smythe H, Phillips R, Dement WC. Quantification of sleepiness: a new approach. Psychophysiology. 1973;10(4):431-6.

3. Hardy CJ, Rejeski WJ. Not what, but how one feels: The measurement of affect during exercise. Journal of Sport & Exercise Psychology. 1989;11(3):304-17.

4. Svebak S, Murgatroyd S. Metamotivational dominance: a multimethod validation of reversal theory constructs. Journal of personality and social psychology. 1985;48(1):107.
